# Estimating Influenza Illnesses Averted by Year-Round and Seasonal Campaign Vaccination for Young Children, Kenya

**DOI:** 10.3201/eid3011.240375

**Published:** 2024-11

**Authors:** Radhika Gharpure, Young M. Yoo, Ben Andagalu, Stefano Tempia, Sergio Loayza, Chiedza Machingaidze, Bryan O. Nyawanda, Jeanette Dawa, Eric Osoro, Rose Jalang’o, Kathryn E. Lafond, Melissa A. Rolfes, Gideon O. Emukule

**Affiliations:** Centers for Disease Control and Prevention, Atlanta, Georgia, USA (R. Gharpure, Y.M. Yoo, B. Andagalu, K.E. Lafond, M.A. Rolfes, G.O. Emukule); Centers for Disease Control and Prevention, Nairobi, Kenya (B. Andagalu, G.O. Emukule); World Health Organization, Geneva, Switzerland (S. Tempia, C. Machingaidze, M.A. Rolfes); Pan American Health Organization, Washington, DC, USA (S. Loayza); Kenya Medical Research Institute, Kisumu, Kenya (B.O. Nyawanda); Washington State University Global Health Kenya, Nairobi (J. Dawa, E. Osoro); Washington State University, Pullman, Washington, USA (E. Osoro); Ministry of Health, Nairobi (R. Jalang’o)

**Keywords:** influenza, viruses, vaccines, respiratory infections, vaccination, child, Kenya

## Abstract

In Kenya, influenza virus circulates year-round, raising questions about optimum strategies for vaccination. Given national interest in introducing influenza vaccination for young children 6–23 months of age, we modeled total influenza-associated illnesses (inclusive of hospitalizations, outpatient illnesses, and non‒medically attended illnesses) averted by multiple potential vaccination strategies: year-round versus seasonal-campaign vaccination, and vaccination starting in April (Southern Hemisphere influenza vaccine availability) versus October (Northern Hemisphere availability). We modeled average vaccine effectiveness of 50% and annual vaccination coverage of 60%. In the introduction year, year-round vaccination averted 6,410 total illnesses when introduced in October and 7,202 illnesses when introduced in April, whereas seasonal-campaign vaccination averted 10,236 (October) to 11,612 (April) illnesses. In the year after introduction, both strategies averted comparable numbers of illnesses (10,831–10,868 for year-round, 10,175–11,282 for campaign). Campaign-style vaccination would likely have a greater effect during initial pediatric influenza vaccine introduction in Kenya; however, either strategy could achieve similar longer-term effects.

Influenza causes a substantial number of respiratory illnesses among young children in Kenya (*1*). The best time to vaccinate against seasonal influenza is before influenza viruses circulate, but because there are multiple periods of increased influenza activity each year in Kenya, the optimum timing and approach for influenza vaccination remains unclear (*2**‒**4*). In 2016, the Kenya National Immunization Technical Advisory Group issued a provisionary recommendation to introduce seasonal influenza vaccination among children 6–23 months of age on the basis of national evidence demonstrating substantial disease in this age group and advised that a pilot vaccination project should be conducted to inform a potential national vaccination rollout (*5*). As a result, a subnational pediatric vaccination pilot was conducted in Kenya during 2019–2021 to compare 2 potential delivery strategies: year-round vaccination, in which influenza vaccine is offered all months of the year, and a once-yearly seasonal vaccination campaign, in which influenza vaccine is offered only for 4 consecutive months (*6*,*7*). Results of the pilot indicated that both strategies might achieve similar vaccine coverage and incur a similar cost per dose for delivery but that the seasonal campaign could require considerable operational needs (e.g., workforce and cold chain capacity) compared with the year-round strategy (*6*,*7*).

In addition to those pilot data on the real-world performance of the proposed strategies, estimating the number of illnesses averted through influenza vaccination can provide additional, relevant evidence to inform pediatric influenza vaccination policy deliberations in Kenya. In other settings, averted illness estimates have provided actionable public health data for influenza vaccine implementation, such as optimizing campaign timing (*8*). In fact, a similar averted illness analysis was previously conducted for pregnant women and young children in Kenya and found that, given the year-round circulation of influenza, both year-round and twice-yearly vaccination strategies might avert a comparable number of illnesses compared with a single annual campaign (*9*). In this analysis, we adapted an existing model (*8*,*10*) to estimate the number of influenza illnesses and hospitalizations averted by influenza vaccine introduction for children 6–23 months of age in Kenya, comparing year-round to seasonal campaign vaccine delivery.

## Methods

### Base Model and Modifications

We adapted a static compartmental model jointly developed by the World Health Organization (WHO), US Centers for Disease Control and Prevention (CDC), and Pan American Health Organization (PAHO) to estimate the number of total influenza-associated illnesses (including hospitalized illnesses, outpatient illnesses, and non‒medically attended illnesses) averted by vaccination. Model methods have been published previously (*8*); in brief, we first estimated the burden of disease among children 6–23 months of age in Kenya in the absence of an influenza vaccination program and then calculated the number of illnesses averted through a counterfactual vaccination program.

We made 3 major modifications to the model described in Chard et al. (*8*) (Appendix Table 1, Figure 1). First, we expanded the time horizon from 12 months to 24 months (as years 1 and 2) to better simulate the effect of residual vaccine protection from the prior year, which was of particular interest for longer-term implementation of year-round vaccine delivery. Second, whereas the original model assumed constant vaccine effectiveness (VE) over the model timeframe, we assumed VE waned in the months after vaccination, as described previously in evaluating influenza vaccination among children and older adults (*11*,*12*). Finally, because we expanded the time period for the new model, we assumed that natural immunity from infection lasted for 12 months, meaning infected persons could recover within the model’s 24-month timeframe and return to the susceptible compartment for their remaining time. In addition, persons infected in the 12 months before the model’s start (year 0) were also systematically returned to the susceptible population as their 12-month immunity expired.

### Vaccination Strategies Modeled

We modeled 2 distinct vaccination strategies: seasonal campaigns, as traditionally used in countries with a Southern or Northern Hemisphere influenza season (*13*), and year-round vaccination, as has been proposed for countries with no clear influenza season (*14*). Although the 2019–2021 demonstration project included campaigns starting in June and July (*6*), we modeled 2 variations of the seasonal campaign with starting months of April (corresponding to the usual timing of Southern Hemisphere influenza vaccine availability) and October (corresponding to Northern Hemisphere vaccine availability) (*13*) (Figure). We assumed each vaccination campaign would last 4 months, as in the demonstration project (*6*). For year-round vaccination, we similarly modeled initial introduction of the vaccine in both April and October for comparability across the 24-month analysis timeframe. As a sensitivity analysis, we included 2 additional start times for the seasonal campaign: June (halfway between Southern and Northern Hemisphere vaccine availability) and January (halfway between Northern and Southern Hemisphere vaccine availability).

**Figure:**
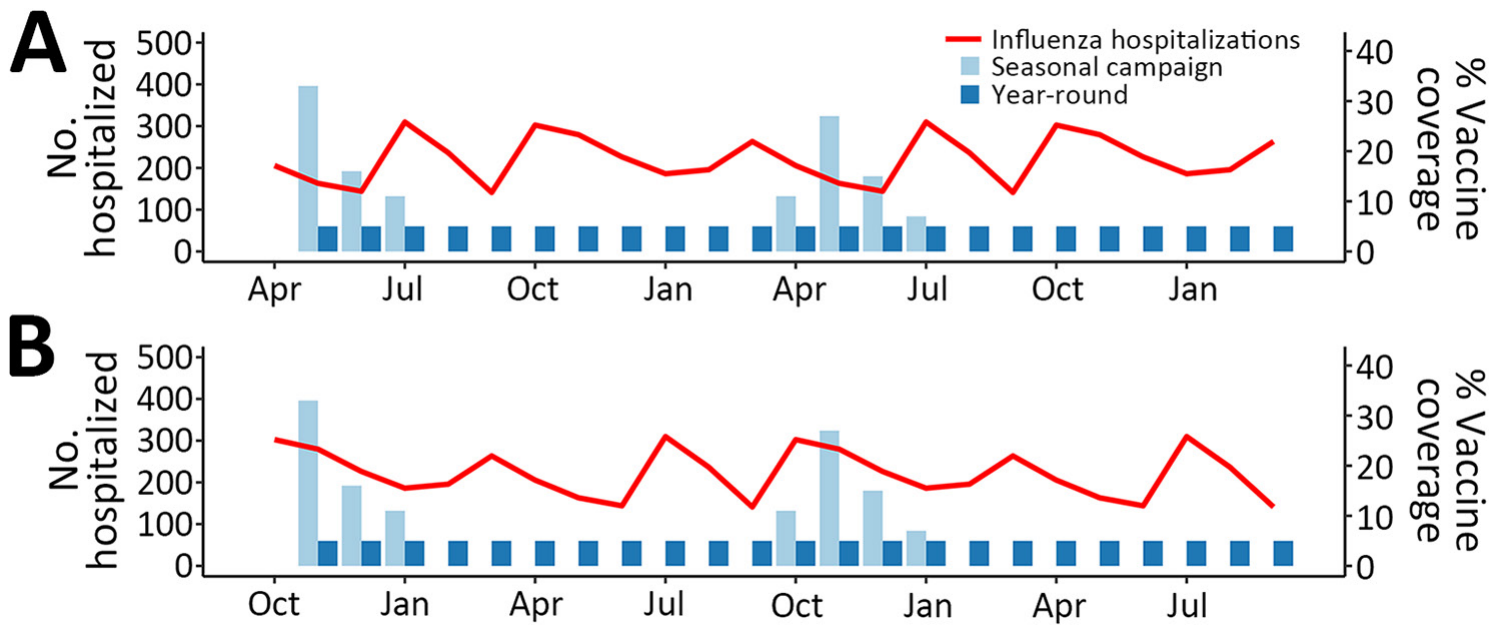
Modeled influenza hospitalizations and vaccine coverage among children 6–23 months of age in Kenya, by delivery strategy. Red line indicates no. monthly hospitalizations, corresponding to the left y-axis. Light blue bars represent percentage vaccine coverage for the seasonal-campaign strategy and dark blue bars the year-round strategy, corresponding to the right y-axis. Hospitalization curves are identical for both the introduction year (months 1–12) and postintroduction year (months 13–24). A) April introduction of vaccination. B) October introduction of vaccination.

### Model Data Inputs

We estimated the 2022 population size for children 6–23 months of age in Kenya (n = 1,809,394) based on growth projections from 2019 census data (*15*,*16*) (Table 1). We used a previously published incidence of influenza-associated severe acute respiratory illness hospitalizations among children 0–23 months of age in Kenya during 2011–2014 (146.6/100,000 population; *17*) to calculate the annual expected number of hospitalizations (n = 2,653). In addition, we derived an average seasonal curve of influenza-associated disease burden from influenza surveillance data reported to the WHO Global Influenza Surveillance and Response System FluNet data platform (*21*) from Kenya for all ages during 2011–2019. Using an aggregate average method (*22*), we calculated a 3-week moving proportion of samples positive for influenza (Appendix Figure 2). We then distributed the 2,653 annual hospitalizations across weeks of the year using this seasonal curve and summed weekly counts to generate monthly hospitalizations; all years of the model (0‒3) used the same hospitalization curve (Figure).

**Table 1:** Model input variables and data sources in study to estimate influenza illnesses averted by year-round and seasonal campaign vaccination for young children, Kenya*

Model input	Value	Data source
Population characteristics		
Population size, children 6–23 mo of age	1,809,394	2022 census projection using 2019 census (*15*) and World Bank annual population growth rates (*16*)
Annual vaccination coverage, %	60	Assumption based on coverage calculated in first year of pediatric demonstration project; both strategies obtained similar coverage (*6*)
Influenza illnesses		
Influenza-associated hospitalization rate	146.6/100,000 population	Rate among children <2 y of age during 2011–2014 (*17*)
Ratio of nonhospitalized to hospitalized influenza illnesses	19.9 (IQR 17.6–21.6)	Calculated from Siaya County SARI surveillance data and healthcare utilization survey (Appendix Figure 3)
Influenza illnesses medically attended, %	47.9	Healthcare utilization survey: results for children <2 y of age in Kenya (*18*)
Vaccine effectiveness and natural immunity		
VE point estimate, %	50 (average)	2010–2012 clinical trial data for trivalent influenza vaccine in children <5 y of age in Kenya (*19*) and meta-analysis of influenza VE among children 6–23 mo of age (*20*)
Rate of waning (base scenario)	VE_t_ = 70 – 1.37 × biweeks + 0.18 × biweeks^2^ – 0.03 × biweeks^3 ^† (≈7% absolute decline per month)	Prior analyses evaluating influenza vaccination timing among older adults (*11*)
Duration of natural immunity from infection	12 mo	Assumption consistent with prior models that did not assume reinfections within a 12-mo time horizon (*8**,**10*)

*VE, vaccine effectiveness. †VE_t_ refers to vaccine effectiveness at time t, calculated based on the number of biweeks (period of 2 weeks) elapsed.

To estimate the number of influenza-associated events by different strata of severity of illness and care-seeking (i.e., hospitalized illnesses, outpatient illnesses, and total illnesses inclusive of non‒medically attended), the model uses multipliers for the ratio of nonhospitalized to hospitalized illnesses and for the proportion of illnesses medically attended. We calculated a multiplier for the ratio of nonhospitalized to hospitalized cases based on a combination of sentinel surveillance data from Siaya County among children 6–23 months of age during 2011–2013 and a 2018 healthcare utilization survey of children <2 years of age in Kenya (*18*) (Appendix Figure 3). We obtained a multiplier for the proportion of illnesses that were medically attended using the same healthcare utilization survey (*18*).

On the basis of results of the demonstration project comparing year-round and campaign vaccine delivery in 2 counties during 2019–2021 (*6*), we assumed that both delivery strategies would achieve equal cumulative vaccine coverage; we set this value to 60% annually for both strategies. We allocated monthly coverage for the year-round strategy evenly across the year and coverage for the seasonal campaign according to the distribution of monthly coverage achieved during the demonstration project (Figure). We assumed that children would receive 2 doses of influenza vaccine >4 weeks apart, in accordance with recommendations for the age group (*23*), to be considered fully vaccinated; thus, no children were considered fully vaccinated at month 1 of vaccine introduction. However, for the first year after introduction (postintroduction year) of the seasonal campaign, we assumed that one third of children who were previously vaccinated would remain in the eligible age group (6–11 months of age in the first year and 18–23 months in the second year), receiving only 1 dose of vaccine and considered fully vaccinated in the first month of the postintroduction year campaign.

For influenza VE inputs, we used an average VE of 50% against any influenza-associated illness, consistent with 2010–2012 clinical trial data for trivalent influenza vaccine in children <5 years of age in Kenya (*19*), as well as a meta-analysis of influenza VE among children 6–23 months of age (*20*). The base scenario incorporated cubic waning of VE as previously described (*11*), with a starting VE of 70% in month 1 and an average VE of 50% over 8 months (Appendix Table 2). We explored 2 additional patterns: scenario A modeled a constant of 50% with vaccine protection lasting 8 months and then 0% VE (no protection) thereafter, and scenario B modeled a constant VE of 50% with protection lasting 12 months (Appendix Table 2).

### Model Outputs

We estimated the number of hospitalizations, outpatient visits, medically attended illnesses (inclusive of both outpatient visits and hospitalizations), and total illnesses (inclusive of medically attended and non–medically attended illnesses) averted by each vaccination strategy and stratified results by year. We also calculated the prevented fraction, defined as the number of illnesses averted by vaccination divided by the number of illnesses in the absence of vaccine. We used Monte Carlo simulation with 5,000 iterations to sample parameter values at random from their distributions and obtained confidence intervals for all estimates. Monte Carlo simulation assumed a Poisson distribution for monthly hospitalizations, a uniform distribution for the ratio of nonhospitalized to hospitalized influenza illnesses, and normal distributions for the proportion of influenza illnesses non‒medically attended, VE, and vaccine coverage. We performed all analyses in R version 4.0.2 (The R Project for Statistical Computing, https://www.r-project.org).

## Results

From the modeled estimates, during the first year that seasonal influenza vaccination was introduced (introduction year), year-round vaccination starting in April averted an estimated 7,202 (95% CI 5,898–8,616) total influenza-associated illnesses in children 6–23 months of age, including 3,450 (95% CI 2,751–4,251) medically attended illnesses and 349 (95% CI 286–416) hospitalizations (Table 2). Year-round vaccination starting in October averted an estimated 6,410 (95% CI 5,218–7,692) total illnesses, including 3,066 (95% CI 2,431–3,769) medically attended illnesses and 311 (95% CI 254–371) hospitalizations (Table 2). The prevented fraction of total illnesses in the introduction year was 13% (95% CI 11%–16%) when year-round vaccination was introduced in April and 12% (95% CI 10%–14%) when introduced in October. In comparison, an April–July (Southern Hemisphere) seasonal campaign averted an estimated 11,612 (95% CI 9,220–14,478) total illnesses, including 5,546 (95% CI 4,287–7,090) medically attended illnesses and 562 (95% CI 448–695) hospitalizations; an October–January seasonal campaign (corresponding to Northern Hemisphere vaccine availability) averted an estimated 10,236 (95% CI 8,082–12,837) total influenza-associated illnesses, including 4,894 (95% CI 3,787–6,286) medically attended illnesses and 497 (95% CI 394–621) hospitalizations. The prevented fraction of total illnesses was 21% (95% CI 17%–26%) for the April–July campaign and 19% (95% CI 15%–23%) for October–January.

**Table 2:** Estimated influenza illnesses averted through influenza vaccination for children 6–23 mo of age in the introduction year (base scenario), Kenya*

Vaccination strategy	Prevented fraction	Hospitalizations averted	Outpatient visits averted	Medically attended illnesses averted	Total illnesses averted
Year-round vaccination, April start	13.2 (10.9–15.7)	349 (286–416)	3,101 (2,456–3,846)	3,450 (2,751–4,251)	7,202 (5,898–8,616)
Year-round vaccination, October start	11.7 (9.6–13.9)	311 (254–371)	2,754 (2,173–3,414)	3,066 (2,431–3,769)	6,410 (5,218–7,692)
Seasonal campaign vaccination, Apr–Jul	21.2 (16.9–26.3)	562 (448–695)	4,988 (3,828–6,391)	5,546 (4,287–7,090)	11,612 (9,220–14,478)
Seasonal campaign vaccination, Oct–Jan	18.7 (14.9–23.3)	497 (394–621)	4,399 (3,377–5,684)	4,894 (3,787–6,286)	10,236 (8,082–12,837)

*Values are estimated no. (95% CI) except for prevented fraction, which was defined as the number of illnesses averted by vaccination divided by the number of illnesses in the absence of vaccine.

In the second year after influenza vaccination was introduced (postintroduction year), year-round vaccination averted an estimated 10,831 (95% CI 8,748–13,106) total illnesses, including 5,188 (95% CI 4,077–6,464) medically attended illnesses and 526 (95% CI 426–635) hospitalizations when introduced in April and 10,868 (95% CI 8,726–13,162) total illnesses, including 5,187 (95% CI 4,079–6,486) medically attended illnesses and 528 (95% CI 425–636) hospitalizations when introduced in October (Table 3). The prevented fraction of total illnesses was 20% (95% CI 16%–24%) for year-round vaccination regardless of the starting month. An April–July seasonal campaign averted an estimated 11,282 (95% CI 8,752–14,099) total illnesses, including 5,392 (95% CI 4,130–6,890) medically attended illnesses and 547 (95% CI 425–684) hospitalizations, and an October–January seasonal campaign averted an estimated 10,175 (95% CI 7,891–12,876) total illnesses, including 4,869 (95% CI 3,703–6,253) medically attended illnesses and 493 (95% CI 384–622) hospitalizations. The prevented fraction was 21% (95% CI 16–26) for the April–July campaign and 19% (95% CI 14–23) for October–January.

**Table 3:** Estimated influenza illnesses averted through influenza vaccination for children 6–23 mo of age in the postintroduction year (base scenario), Kenya*

Vaccination strategy	Prevented fraction	Hospitalizations averted	Outpatient visits averted	Medically attended illnesses averted	Total illnesses averted
Year-round vaccination, April start	19.8 (16.1–23.9)	526 (426–635)	4,656 (3,640–5,831)	5,188 (4,077–6,464)	10,831 (8,748–13,106)
Year-round vaccination, October start	19.9 (16.1–23.9)	528 (425–636)	4,658 (3,635–5,861)	5,187 (4,079–6,486)	10,868 (8,726–13,162)
Seasonal campaign vaccination, Apr–Jul	20.6 (16.1–25.7)	547 (425–684)	4,845 (3,697–6,221)	5,392 (4,130–6,890)	11,282 (8,752–14,099)
Seasonal campaign vaccination, Oct–Jan	18.6 (14.4–23.4)	493 (384–622)	4,371 (3,308–5,638)	4,869 (3,703–6,253)	10,175 (7,891–12,876)

*Values are estimated no. (95% CI) except for prevented fraction, which was defined as the number of illnesses averted by vaccination divided by the number of illnesses in the absence of vaccine.

Sensitivity analyses indicated that assuming a constant 8-month VE without waning (scenario A) did not significantly change the estimate of averted illness for any strategy in the introduction or postintroduction years compared with the base scenario (Appendix Table 3, Figure 4). Assuming a constant 12-month VE (scenario B) increased the absolute number of averted illnesses for all strategies in the postintroduction year, but 95% CIs overlapped with the base scenario for the April seasonal campaign and year-round strategy with October introduction (Appendix Table 4, Figure 4). In addition, sensitivity analyses that changed the starting month of the seasonal campaign to January or June did not significantly affect the estimates of averted illnesses (Appendix Figure 5).

## Discussion

Introducing influenza vaccination for children 6–23 months of age in Kenya would reduce the number of influenza-associated illnesses substantially. Our results indicate that campaign-style vaccination would likely have the greatest effect for initial introduction because it would most rapidly achieve high population protection in a vaccine-naive population. Our finding is consistent with recommendations for catch-up campaigns, or initial campaign-style rollout of vaccination to accelerate herd protection, for other vaccines; for example, modeling findings indicated that catch-up campaigns would increase the efficiency of pneumococcal conjugate vaccine introduction for children <5 years of age in Kenya (*24*). However, our results suggest that, after the initial introduction, either year-round vaccination or a seasonal campaign could have similar effects for long-term implementation. Therefore, national policy decisions regarding which strategy to implement for influenza vaccination should consider the full portfolio of evidence available from the demonstration project, including coverage, costs, effects on the wider health system, logistical feasibility, and perceptions from both the community and health workers (*6*,*7*,*25*). For example, implementation experiences from the vaccination pilot suggested greater operational demands within a short time for the campaign strategy (*6*), but year-round delivery could also be operationally complex (*9*,*14*); previously proposed options include using both Northern and Southern Hemisphere formulations during their respective influenza seasons or extending use of a single formulation for the entire year (*14*). More data are needed on clinical protection offered by those options and programmatic feasibility of changing formulations during a year to inform how year-round vaccination would be implemented. Prior analyses have also considered a twice-yearly vaccination campaign strategy which might offer an intermediate option to year-round and single annual vaccination (*5*,*9*); however, we did not include that strategy in this analysis for comparability with the 2019–2021 demonstration project (*6*).

The first limitation of our study is that the results from our analysis are greatly dependent on the 2011–2019 influenza seasonal curve that we calculated using FluNet data for Kenya. Because the seasonal curve we calculated from surveillance data showed no clear single seasonal peak, our results indicated that timing a vaccination program with either Southern or Northern Hemisphere vaccine availability might have similar results in Kenya. Previous analyses characterizing influenza seasonality in Kenya are not consistent; a recent analysis suggested that Kenya had 3 influenza seasons, occurring primarily during the Southern Hemisphere seasonal months (*19*), whereas others have suggested a primarily Northern Hemisphere epidemic (*26*), 2 major epidemics (*3*), or unascertainable seasonality (*4*). Even within the years included in the seasonal curve calculation, not all matched the average seasonality exactly; some years have larger peaks at different times, and, thus, use of a seasonal curve does not account for potential year-to-year variations in incidence or temporal distribution of influenza illnesses, which could influence the model results. In addition, to calculate the annual number of influenza hospitalizations, we used an estimate of incidence among children 0–23 months of age during 2011–2014, which might not be representative of all years; because incidence among children 0–5 months of age might be slightly lower than incidence for those 6–11 and 12–23 months (*17*), inclusion of this age group might have underestimated true incidence among the target population 6–23 months of age. Similarly, the results are dependent on the VE estimate used, which can also vary year-to-year based on the level of vaccine match with circulating viruses (*23*). We assumed the same VE for both the Northern and Southern Hemisphere vaccine formulations, as well as for both severe illness (i.e., hospitalization) and mild illness, which might in reality have differential VEs (*27*).

Second, we based our models on assumptions about duration of vaccine-induced immunity, which we modeled from earlier analysis of VE among older adults rather than young children. However, analyses have indicated similar patterns of waning of VE among children and older adults (*12*), and sensitivity analyses using a constant VE suggested that the base scenario might, in fact, be a conservative estimate of the vaccine’s impact. Similarly, we assumed that previous infection would confer 12 months of natural immunity, consistent with earlier models that did not include reinfections within a 12-month timeframe (*8*,*10*). Cohort studies have indicated that reinfections with influenza virus are possible within a single season (*28*,*29*); thus, our model assumptions might not fully reflect the duration of natural immunity and likelihood of reinfection in Kenya, particularly given year-round influenza circulation. Third, given the relatively short timeframe examined (24 months), we assumed a stable population and did not account for potential changes in population size (e.g., births, deaths, and migrations) within the model period. Fourth, results in the postintroduction year include residual immunity from the previous year. If a catch-up campaign were used for initial vaccine introduction and the program transitioned to year-round vaccination, the effect of the vaccine would not be equivalent to the postintroduction year results until >2 years of year-round vaccination had occurred. Fifth, we assumed that both strategies would achieve the same annual vaccine coverage based on results of the vaccination pilot (*6*), but it is possible that a year-round strategy could reach more children during noncampaign months and result in higher coverage, as previously modeled for maternal influenza vaccination (*9*), or that differences in vaccination program costs could result in differential coverage achieved (*7*).

Finally, we assumed that vaccinated persons were either fully protected by their vaccine or unprotected. Alternative assumptions—for example, where all vaccinees experience a reduced rate of acquiring infection—might result in a higher estimated number of illnesses prevented by vaccination (*30*). In addition, the model did not include nonrespiratory manifestations of influenza illness (*31*), did not account for partial immunity received from a single dose of influenza vaccine among young children (*23*,*32*), and did not include indirect effects of the vaccine (*33*) or estimates of averted deaths (*34*). Furthermore, because less than one third (32%) of young children with severe pneumonia were hospitalized in a previous healthcare utilization survey in Kenya (*20*), a substantial burden of severe illness is missed by focusing on hospitalizations alone. Therefore, our results likely underestimate the full value and effect of influenza vaccination, which warrant further evaluation.

Our findings indicate that either year-round or seasonal campaign vaccination for influenza could substantially reduce influenza burden among children 6–23 months of age in Kenya, resulting in health and economic benefits to children and their families, as well as to the health system and broader society. The results of our analysis suggest that campaign-style vaccination would achieve the greatest impact for in the first year of introduction; however, longer-term implementation could employ either strategy to achieve similar effect. Our data can be used to inform additional analyses, such as estimating averted cost burden, cost-benefit, or cost-effectiveness of vaccination. Policy decisions regarding the most appropriate vaccination strategy should holistically weigh the available evidence and local implementation context.

Appendix Additional information about estimating influenza illnesses averted by year-round and seasonal campaign vaccination for young children, Kenya.
