# Supplementary material for: Estimating Influenza Illnesses Averted by Year-Round and Seasonal Campaign Vaccination for Young Children, Kenya
Source: Emerg Infect Dis. 2024 Nov;30(11):2362–9. doi: 10.3201/eid3011.240375 (PMC11521166; doi:10.3201/eid3011.240375)
Supplement: Appendix — Additional information about estimating influenza illnesses averted by year-round and seasonal campaign vaccination for young children, Kenya. [file 24-0375-Techapp-s1.pdf]

Article DOI: http://doi.org/10.3201/eid3011.240375

EID cannot ensure accessibility for supplementary materials supplied by authors. Readers who have difficulty accessing supplementary content should contact the authors for assistance.

Estimating Influenza Illnesses Averted by Year-Round and Seasonal Campaign Vaccination for Young Children, Kenya

Appendix

Appendix Table 1. Input parameters and equations for the modified compartmental model describing the transmission dynamics of an influenza virus in a population with vaccination

Step	Variable name	Variable label	Equation
1	Pop	Population at risk, excluding initial number of individuals with natural infection immunity	Input data
2	$Case_m$	Observed cases between month m-1 and m	Input data
3	NS_m	Individuals with natural infection immunity returning to susceptible population between month m-1 and m[1]	Input data
4	Sn_m	Susceptible population in month m in the absence of vaccine	$Sn_{m-1} - Case_m + Case_{m-12\ if\ m>12} + NS_m$
5	λ_m	Incidence rate between month m-1 and m	$Case_m / Sn_{m-1}$
6	γ	Recovery rate[2]	Input data
7	VC_m	Vaccine coverage between month m-1 and m	Input data
8	VE	Vaccine effectiveness	Input data
9	V_m	Effectively vaccinated population between month m-1 and m	$Pop \cdot VC_m \cdot VE$
10	$RS_{1,m}$	Individuals returning to susceptible population due to loss of vaccine-induced immunity between month m-1 and m[3]	$\sum_{i=1}^{m-1} Pop \cdot VC_i \cdot [(VE - 1.37 \cdot 2 \cdot (\mathrm{m}-1-\mathrm{i}) + 0.18 \cdot 2^2 \cdot (m-1-i)^2 - 0.03 \cdot 2^3 \cdot (m-1-i)^3) - (VE - 1.37 \cdot 2 \cdot (\mathrm{m}-\mathrm{i}) + 0.18 \cdot 2^2 \cdot (m-i)^2 - 0.03 \cdot 2^3 \cdot (m-i)^3)]$
11	$RS_{2,m}$	Individuals returning to susceptible population due to loss of infection-induced immunity between month m-1 and m	$I_{m-12\ if\ m>12} + NS_m$
12	S_m	Susceptible population in month m in the presence of vaccine	$S_{m-1} - \lambda_m S_{m-1} - V_m + RS_{1,m} + RS_{2,m}$
13	I_m	Estimated cases in month m in the presence of vaccine	$I_{m-1} + \lambda_m S_{m-1} - \gamma I_{m-1}$
14	R_m	Immunized population in month m in the presence of vaccine (e.g., effectively vaccinated or recovered after natural infection)	$R_{m-1} + \gamma I_{m-1} + V_m - RS_{1,m} - RS_{2,m}$
15	$Avert_m$	Averted cases	$Case_m - I_m$
16	$Frac_m$	Prevented fraction	$Avert_m / Case_m$

Note: subscripts indicate month, i.e., "m" indicates current month, "m-1" indicates prior month
[1] Refers to individuals who were infected before the start of the model timeframe (e.g., at year 0), with the assumption that natural immunity lasts for 12 mo, leading them to return to susceptible population thereafter
[2] We assumed that all individuals were infected in month m-1 and were recovered/immunized in month m
[3] In cases where VE decreased to a negative value, we assumed that the individual no longer had vaccine-induced immunity, and therefore set VE to 0%

Appendix Table 2. Description of sensitivity analyses

Assumptions	Base scenario	Alternate scenarios	
		Scenario A	Scenario B
Vaccine effectiveness	Cubic decay with starting VE of 70%	Constant VE for 8 mo with starting VE of 50%	Constant VE for 12 mo with starting VE of 50%
VE: month 1	70%	50%	50%
VE: month 2	67.7%	50%	50%
VE: month 3	65.5%	50%	50%
VE: month 4	61.8%	50%	50%
VE: month 5	52.2%	50%	50%
VE: month 6	44.3%	50%	50%
VE: month 7	27.6%	50%	50%
VE: month 8	3.8%	50%	50%
VE: month 9	0%	0%	50%
VE: month 10	0%	0%	50%
VE: month 11	0%	0%	50%
VE: month 12	0%	0%	50%

Appendix Table 3. Estimated influenza illnesses averted through influenza vaccination for young children in Kenya, by delivery strategy and month of introduction (Scenario A; 8-mo constant vaccine effectiveness)

	Introduction year, n (95% CI)					Postintroduction year, n (95% CI)				
Strategy	Prevented fraction*	# hospitalizations averted	# outpatient visits averted	# medically attended illnesses averted	# total illnesses averted	Prevented fraction[1]	# hospitalizations averted	# outpatient visits averted	# medically attended illnesses averted	# total illnesses averted
Year-round vaccination (beginning in April)	11.6 (9.2–14.1)	308 (244–374)	2,730 (2,094–3,439)	3,039 (2,341–3,805)	6,343 (5,012–7,735)	20.1 (16.1–24.1)	532 (425–640)	4,715 (3,650–5,883)	5,252 (4,076–6,513)	10,957 (8,724–13,241)
Year-round vaccination (beginning in October)	10.4 (8.3–12.6)	276 (218–336)	2,448 (1,885–3,085)	2,725 (2,114–3,416)	5,700 (4,484–6,962)	20.1 (16.1–24.2)	534 (424–642)	4,712 (3,646–5,916)	5,250 (4,087–6,544)	10,998 (8,715–13,298)
Seasonal campaign vaccination (April–July)	21.2 (16.7–26.1)	561 (442–693)	4,964 (3,791–6,371)	5,523 (4,252–7,051)	11,562 (9,067–14,383)	20.6 (15.9–25.8)	548 (421–684)	4,850 (3,663–6,229)	5,397 (4,093–6,894)	11,309 (8,658–14,129)
Seasonal campaign vaccination (October–January)	19.4 (15.3–24)	515 (405–642)	4,559 (3,477–5,858)	5,073 (3,894–6,476)	10,611 (8,318–13,214)	19 (14.6–23.7)	503 (387–632)	4,454 (3,354–5,743)	4,959 (3,749–6,360)	10,367 (7,952–13,061)

*The prevented fraction was defined as the number of illnesses averted by vaccination divided by the number of illnesses in the absence of vaccine.
Abbreviations: CI, confidence interval

Appendix Table 4. Estimated influenza illnesses averted through influenza vaccination for young children in Kenya, by delivery strategy and month of introduction (Scenario B; 12-mo constant vaccine effectiveness)

	Introduction year, n (95% CI)					Postintroduction year, n (95% CI)				
Strategy	Prevented fraction*	# hospitalizations averted	# outpatient visits averted	# medically attended illnesses averted	# total illnesses averted	Prevented fraction[1]	# hospitalizations averted	# outpatient visits averted	# medically attended illnesses averted	# total illnesses averted
Year-round vaccination (beginning in April)	12.3 (9.8–14.9)	327 (259–398)	2,902 (2,224–3,657)	3,229 (2,489–4,041)	6,739 (5,319–8,227)	30.1 (24.1–36)	796 (636–956)	7,058 (5,466–8,806)	7,854 (6,112–9,759)	16,417 (13,110–19,787)
Year-round vaccination (beginning in October)	10.9 (8.7–13.3)	291 (229–354)	2,571 (1,980–3,242)	2,864 (2,215–3,589)	5,986 (4,701–7,311)	30 (24–36)	796 (635–956)	7,034 (5,450–8,818)	7,833 (6,101–9,762)	16,409 (13,022–19,760)
Seasonal campaign vaccination (April–July)	24.9 (19.7–30.6)	660 (520–816)	5,856 (4,470–7,497)	6,515 (4,997–8,289)	13,620 (10,695–16,862)	30.6 (24–37.7)	813 (634–1004)	7,199 (5,494–9,151)	8,012 (6,145–10,121)	16,760 (13,080–20,676)
Seasonal campaign vaccination (October–January)	22.3 (17.6–27.6)	591 (466–734)	5,233 (3,995–6,703)	5,821 (4,470–7,424)	12,169 (9,531–15,169)	30.9 (24.3–37.9)	819 (643–1011)	7,266 (5,567–9,205)	8,085 (6,221–10,184)	16,883 (13,228–20,873)

*The prevented fraction was defined as the number of illnesses averted by vaccination divided by the number of illnesses in the absence of vaccine.
Abbreviations: CI, confidence interval

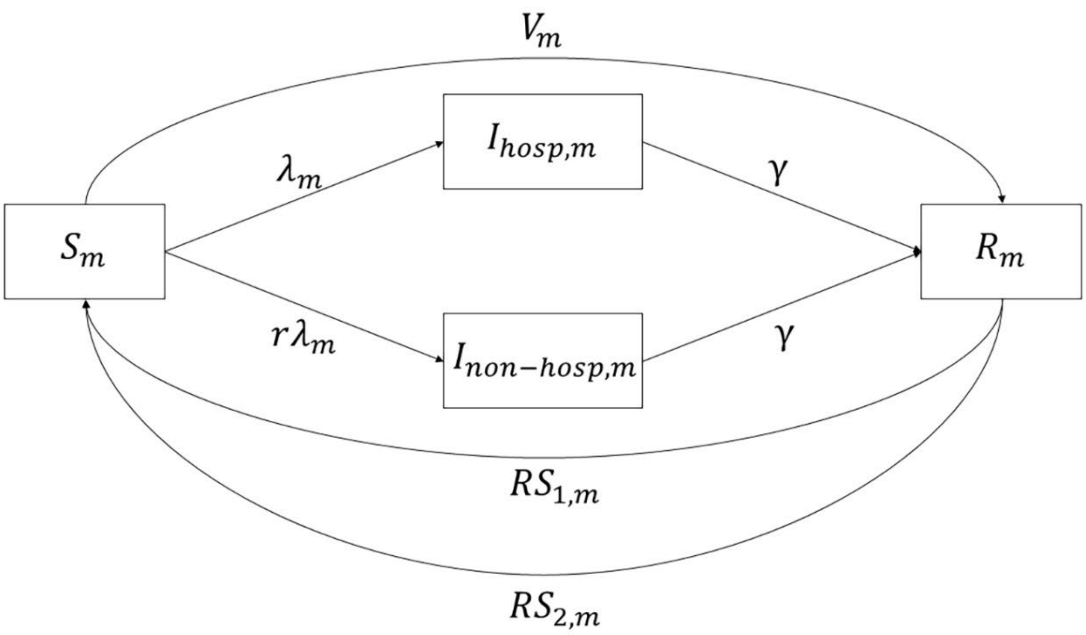

Sensitivity: Official Use

Appendix Figure 1. Structure of the modified compartmental model describing the transmission dynamics of an influenza virus in a population with vaccination

S_m: susceptible population in month m

$I_{hosp,m}$: number of hospitalized cases in month m

$I_{nonhosp,m}$: number of nonhospitalized cases in month m

R_m: number of individuals effectively vaccinated or recovered in month m

λ_m: incidence rate of hospitalizations in month m

r: multiplier for ratio of nonhospitalized cases to hospitalized cases

γ: recovery rate

V_m: number of individuals effectively vaccinated in month m

$RS_{1,m}$: number of individuals returning to susceptible population due to loss of vaccine-induced immunity in month m

$RS_{2,m}$: number of individuals returning to susceptible population due to loss of infection-induced immunity in month m

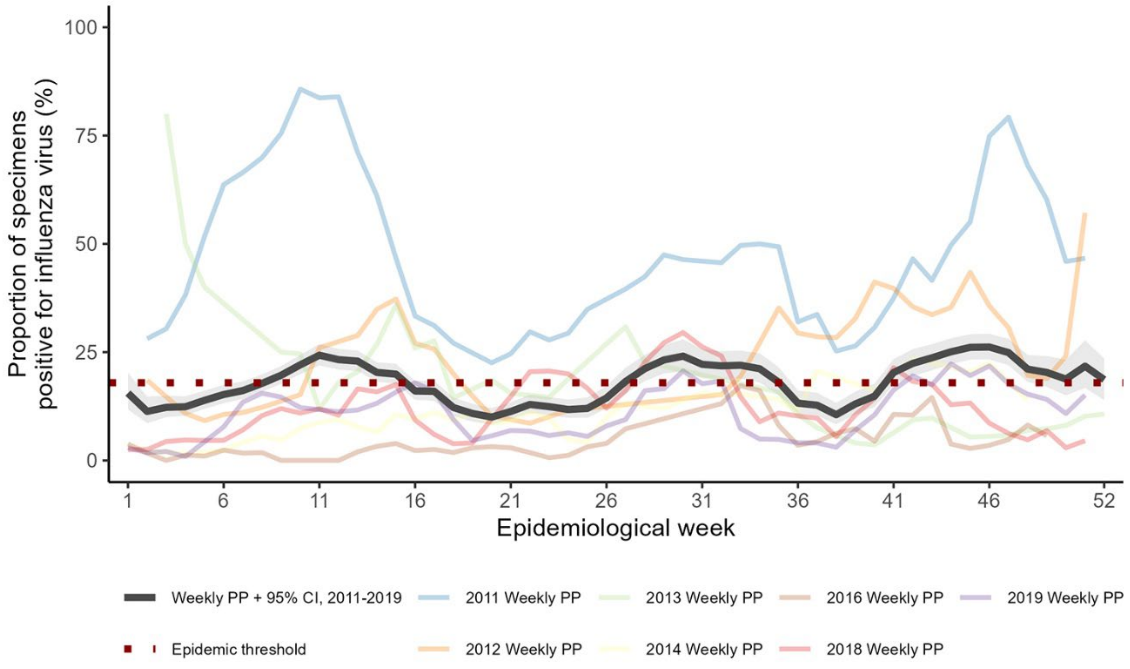

Appendix Figure 2. Average weekly percent positivity derived from the WHO Global Influenza Surveillance and Response System (GISRS) FluNet data platform for Kenya, 2011–2019.

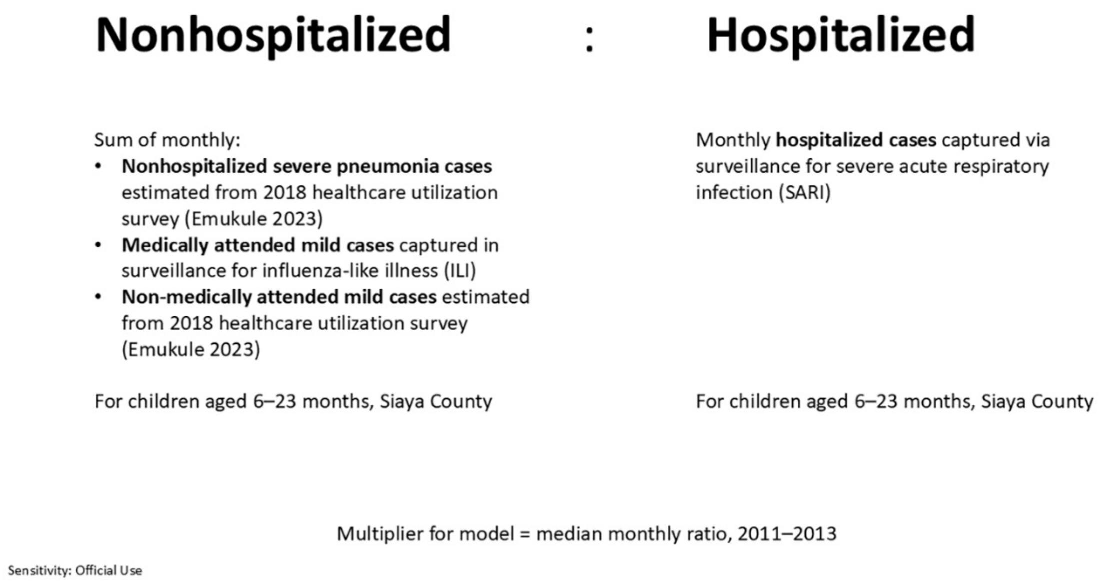

Appendix Figure 3. Calculation of multiplier for the ratio of nonhospitalized to hospitalized illnesses.

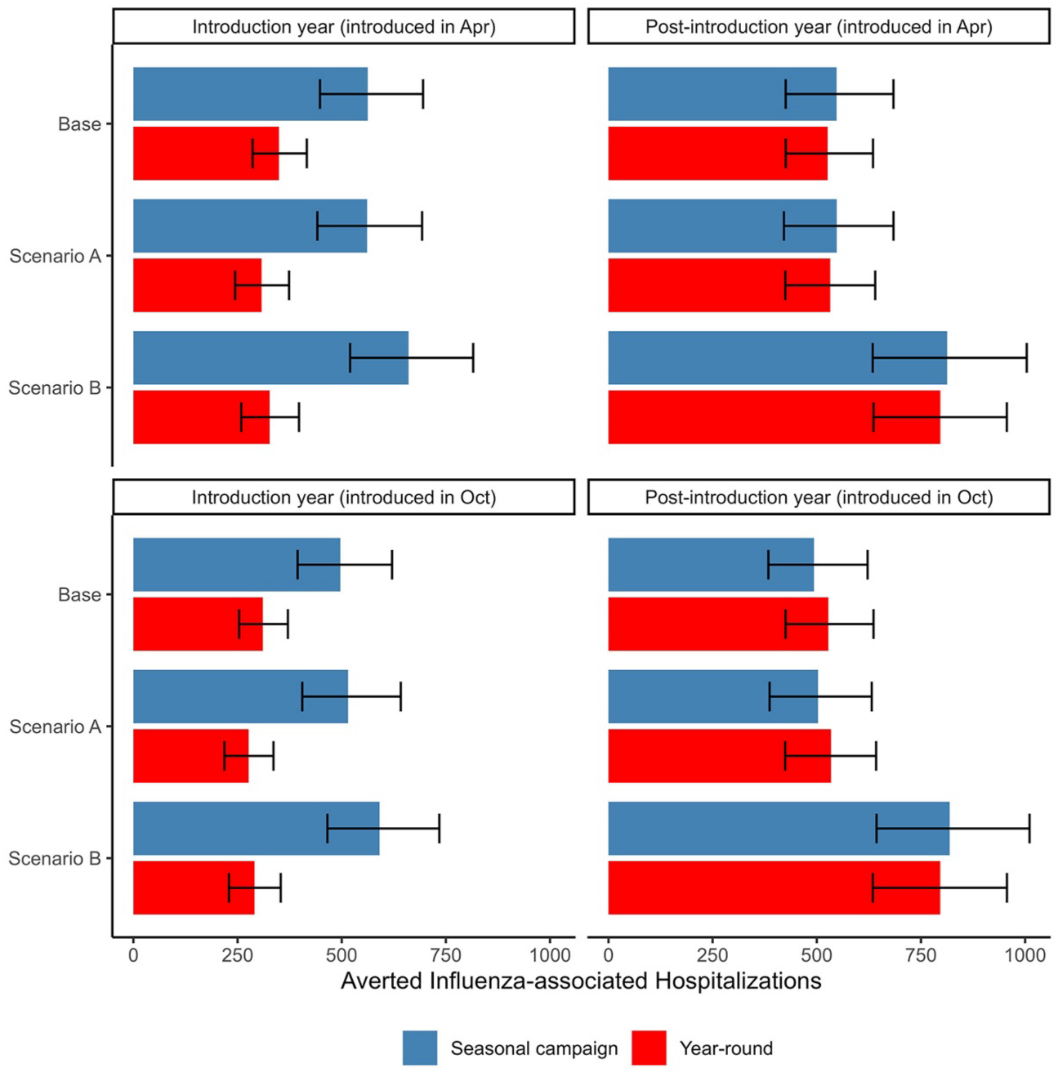

Appendix Figure 4. Estimated influenza illnesses averted through influenza vaccination for young children in Kenya, under base scenario and alternate vaccination scenarios.

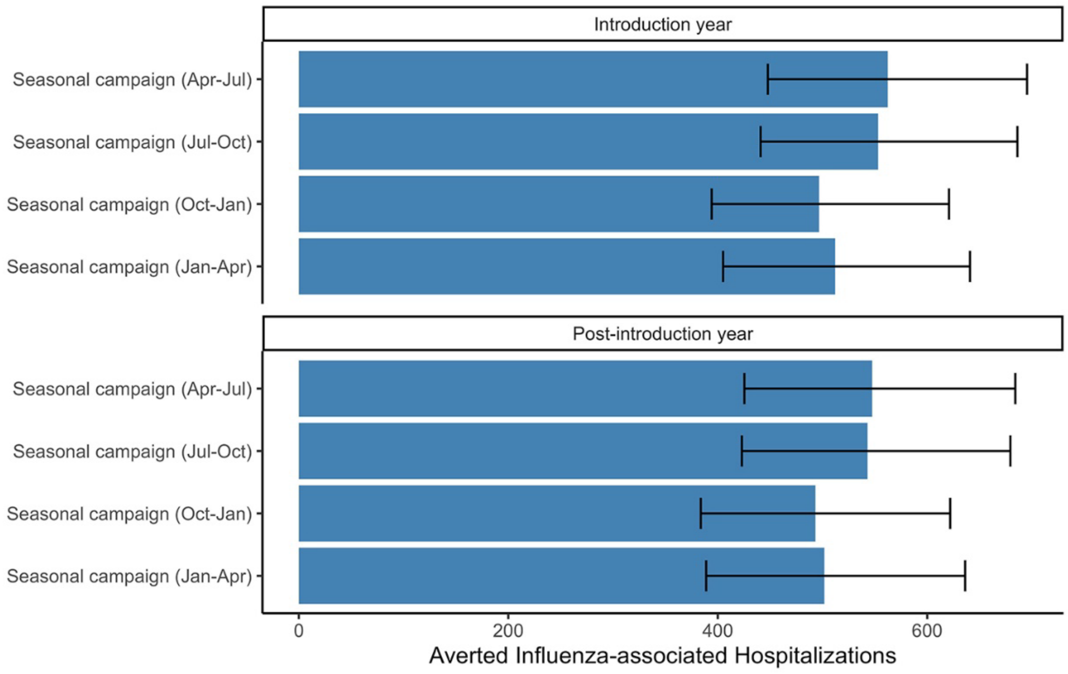

Appendix Figure 5. Estimated influenza illnesses averted through influenza vaccination for young children in Kenya for seasonal campaigns beginning in April, June, October, and January.
